# Recent insights in the correlation between social media use, personality traits and exercise addiction: a literature review

**DOI:** 10.3389/fpsyt.2024.1392317

**Published:** 2024-05-10

**Authors:** Adele Minutillo, Annagiulia Di Trana, Valeria Aquilina, Gerolama Maria Ciancio, Paolo Berretta, Nunzia La Maida

**Affiliations:** ^1^ National Centre on Addiction and Doping, Italian National Institute of Health, Rome, Italy; ^2^ Università degli Studi Internazionali di Roma, Rome, Italy

**Keywords:** exercise addiction, body image, perfectionism, behavior, addictive

## Abstract

**Introduction and aim:**

The excessive involvement in physical activity without stopping in between sessions despite injuries, the continuous thinking to exercise feeling insane thoughts and experiencing withdrawal symptoms are all characteristics of the Exercise Addiction (EA), an addictive behavior. While the primary exercise addiction is directly caused by compulsive exercise, many studies highlighted the relationship between Eating Disorders (ED) and EA, defining the secondary EA. The correlation between EA, social media use (SMU) and other individual traits remains a relatively underexplored domain. Therefore, this review aimed to examine the latest evidence on the relationship between EA, SMU, and some personality traits such as perfectionism and body image.

**Methods:**

Electronic databases including PubMed, Medline, PsycARTICLES, Embase, Web of Science were searched from January 2019 to October 2023, following the PRISMA guidelines.

**Results:**

A total of 15 articles were examined and consolidated in this review. EA was found to be related to different individual traits such perfectionism, body dissatisfaction, depression, obsessive-compulsive personality disorders. While controversial results were found regarding the relationship between EA and SMU.

**Conclusion:**

The interaction between mental health, exercise addiction and social media use is complex. Excessive engagement in these latter may result in negative mental health consequences despite their potential benefits. Understanding individual differences and developing effective interventions is crucial to promoting healthy habits and mitigating the EA risks, ultimately enhancing mental well-being. Further research should focus on the identification of risks and protective factors with the eventual aim of developing and implementing effective prevention strategies.

## 1 Introduction

The constant pursuit of a healthy lifestyle is widely related to the growing attention to physical and mental health to contrast the acceleration of the societal ageing process. Whereas physical exercise and sports engagement were widely valued, excessive involvement in exercise may drive to addictive behavior, referred to as exercise addiction (EA). Although not formally recognized in diagnostic manuals such as the Diagnostic and Statistical Manual of Mental Disorders 5 (1), EA, also known as “exercise dependence” or “compulsive exercising”, is acknowledged as a behavioral addiction. Indeed, the discrimination between exercise-addicted and regular exercisers is challenging, making the symptom intensity evaluation of particular importance (2).

The uncontrollable urge to engage in physical activity that surpasses health or fitness requirements is the main characteristic of this addiction. Conversely to regular and healthy physical exercise, excessive engagement may lead to adverse consequences. Adverse effects include tolerance and withdrawal symptoms, mood alteration, impulsivity, lack of control, detrimental social and financial consequences, physical injuries (3, 4). Specifically, when the exercise’s positive effects on mood and well-being are transient, it may exert a feeling of deprivation when exercise is inaccessible, a compulsion to resume exercise promptly, negative emotions, increased exercise duration, inability to cease exercise even when injured, and sleeplessness (5, 6). Furthermore, secondary EA was recently defined as an aspect of eating disorders (ED), characterized by obsessive exercising in conjunction with anorexia or bulimia nervosa (7). In this case, a body image disturbance may exist at the base of the EA, besides heightened levels of anxiety and depression (8). The pursuit of physical perfection and fixation on maintaining a specific body image may contribute to the onset of EA, detrimentally impacting mental well-being (9, 10). Problematic Social media use (PSMU) and ED, like EA, are linked to several psychological and physical health problems including difficulties in emotion regulation, psychological distress, excessive daytime sleepiness and body dissatisfaction (11, 12). Some studies suggest that individuals with EA may be inclined more towards using social media to showcase their fitness achievements, seeking validation from online peers (13–15). These individuals may be trapped by the carefully curated nature of social media content, depicting unattainable representations of individuals’ lives, affecting their self-esteem (13, 16).

Moreover, a relationship between EA, PSMU or social media addiction (SMA), and perceived discomfort regarding images of physical idealization was corroborated by the so-called “fitspiration” (17–19). This term derives from the fusion of “fitness” and “inspiration,” which involves posting online, primarily through social networking channels, images promoting health, wellness, healthy eating, self-care, and especially physical exercise (20). Moreover, the “fear of missing out” (FOMO) phenomenon on social media could drive individuals to excessively engage in both exercise and social media use, contributing to adverse mental health outcomes (17, 21).

Conversely, excessive exposure to fitness-related content on social media might exacerbate exercise addiction by perpetuating unrealistic body standards and nurturing an obsession with exercise (22, 23).

The main focus of studies reported in the literature is on defining, diagnosing, characterizing, and elucidating comorbidities (16, 24). Moreover, current researches examine the relationship among EA ED and anxiety (25–27) making the specific correlation between EA, SMU and other personality traits (perfectionism, body image) still a domain that has not yet been fully explored. For this reason we performed a literature review to clarify the relationship between EA, SMU, and mental health outcomes by bringing together existing research and examining the underlying mechanisms that drive their interactions.

## 2 Methods

**Figure 1:**
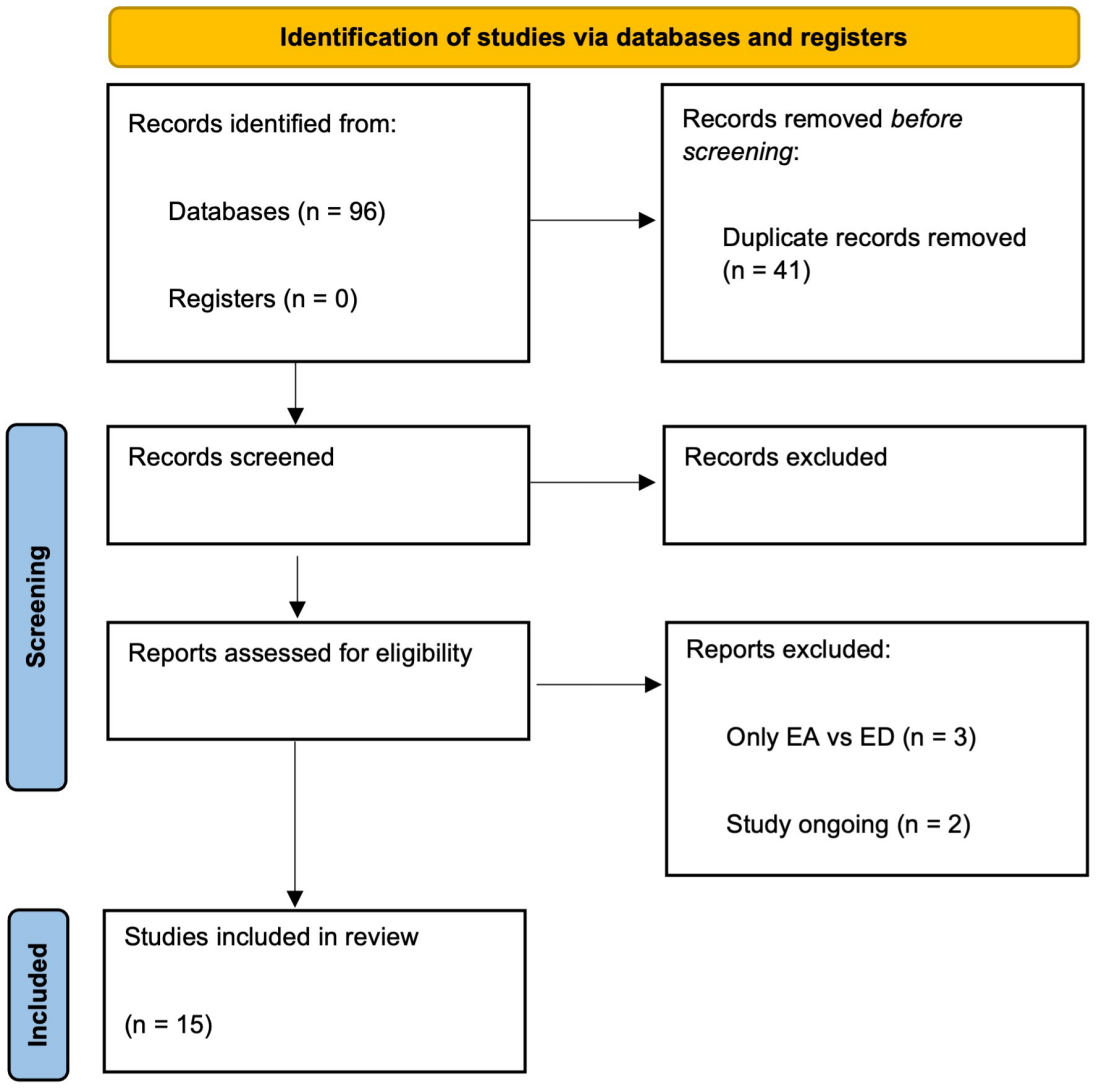
PRISMA flow diagram of the study selection.

We performed comprehensive literature research to identify articles investigating the relationship between exercise addiction, social media use, and personality traits. Considering the recent focus on the EA-related issues, Electronic databases including PubMed, Medline, PsycARTICLES, Embase, Web of Science were searched from January 2019 to October 2023. Preferred reporting items for systematic reviews and meta-analysis (PRISMA) statement was the methodology selected for the present review (28). According to guidelines of the 2020 PRISMA statement (29) the research team evaluated the following items: definition of the research question, hypothesis and objectives; bibliographic search; data collection, screening of the scientific papers selected and finally, analysis of the main findings and conclusions including the strengths and weakness of these studies (**Figure 1**). Our eligibility criteria included: articles written in English, cross-sectional, longitudinal, and case control studies investigating the association between exercise addiction, social media use and individual traits (e.g. perfectionism, perceived body image, depression), original research performed in general population, adolescents or professional athletes, studies using reliable research tools. Papers published in non-English languages were excluded. Reviews were also excluded but were used for the snowball search strategy. The researchers and performed the initial selection of original manuscripts by screening titles and abstracts, creating a reference list of papers for the topics evaluated in the present review using Rayyan software (30). Two investigators conducted each stage of the studies selection, deleted duplicate inputs and reviewed studies as excluded or requiring further assessment. All data were extracted by two investigators and cross-checked by the other investigator. In case of discrepancies in the selected studies, we opted for reconciliation through team discussion. This narrative review protocol was registered in PROSPERO (international prospective register of systematic reviews) on the 7^th^ of February 2024, with the registration number CRD42024510767.

## 3 Results

### 3.1 Literature research

A total of 96 studies were identified from the initial search, of which 41 duplicates were removed. Titles and abstracts of the rest 55 studies were screened according to the predefined inclusion criteria, and 35 studies were excluded. In total, 15 articles were critically reviewed and consolidated for this review (**Table 1**). The studies were mainly conducted in the last 2 years (2022 and 2023), with prevalence in Australia (n = 5) and Europe (n = 6). The considered population was more often specific, such as gym instructors, competitive athletes, or clinical populations (individuals with ED).

**Table 1:** Studies investigating the relationship between exercise addiction, social media use, and personality traits.

Authors, year country	Study design and objectives	Domain investigated	Sample	Tools	Key findings
Hauck et al., 2019 (31) Germany	Cross-sectional study to investigate the relationships among EA, perfectionism and EA	FA, EA, perfectionism	Participants: 1,022 ♂446 ♀576 Age: 36y.o. (average)	YFAS 2.0, MIPS, FESA	30.5% EA 6.2% FA Perfectionism associated with EA and FA EA mediates the relationship between perfectionism and FA in vulnerable athletes
Freire et al., 2020 (32) Brazil	Transversal descriptive study to investigate the association between body dissatisfaction, EA and risk behavior to eating disorders	Body dissatisfaction, ED, EA	Participants: Fitness: 40 Crossfit: 16, Age: 26.6y.o. (average)	EAT-26, ORTO-15, BSQ, SDE	80% orthorexic behavior and had low degree of EA 30% BD (♀ prevalent) 41.7% ED, age and type of exercise influence BD and EA, positive and weak correlation. Significant correlation according to path analysis model (8% variance)
Rankin et al., 2021 (33) Australia	Cross-sectional study to assess if EMS and negative implicit self-esteem were predictive of self-reported EA behavior.	EMS domains: disconnection and rejection; impaired autonomy and performance; impaired limits; other-directedness; hyper-vigilance and inhibition.	Participants: Cyclists: 136 Age: 18–65y.o (range), 45.8y.o. (average)	YSQ-S3, EDS-21, SE-IAT	Positive prediction of EA by EMS, domain “other directedness” and “impaired limits” no significant relationship between implicit self-esteem and EA
Meyer et al., 2021 (34) Switzerland	Cross-sectional study to identify comorbidities in individuals with EA	Depression. Personality disorders: obsessive-compulsive, narcissistic, antisocial, borderline and histrionic behaviours. Anxiety, ED, substances use, disruptive disorders.	Participants: 32 ♂16 ♀16 Age: 27.9y.o (average)	EDS-21, SAPAS, SCID-5	56.3% depressive disorders 46.9% personality disorders 31.3% obsessive-compulsive disorders. mental disorders number is proportional to the EA severity.
Akbari et al., 2022 (35) Iran/United Kingdom	Investigation on EA relationship between PSMU and negative mental health consequences	PSMU, EA, anxiety, depression, stress, insomnia, body image concern, ED	Participants: 1,307 ♀562 Age: 15y.o. (average) ♂745 Age: 26y.o. (average) recruiting on the most popular social media apps	BSMAS, EAI, EAI-Y, BICI, CES, DASS-21, ISI	EA risk prevalence: 2.7% adolescents 4.4% for young adults. Strongest correlation: - psychological distress and body image concern (adolescents) - EA and compulsive eating (among young adults) direct effect for PSMU on EA, insomnia, anxiety, depression, stress, body concern and compulsive eating. - EA significant mediator in the relationship between PSMU and all mental health outcomes.
Colledge et al, 2022 (36) Switzerland, Germany	Assessment of co-occurance of EA risk with depression and ADHD	ADHD, depression, EA	Participants: 123 ♂ 80 ♀43 Age: 18–70 (range) No random sampling	EDS, CTQ, BDI, HASE	previous diagnosis of anxiety disorder correlated to EA
Cresswell et al., 2022 (37) Australia	Investigation on perfectionism direct and indirect association with ED symptoms through compulsive exercise	Adolescent, compulsive exercise, ED, perfectionism,	Participants: 149 with eating disorders ♀149 Age: 13–17y.o.(range), 14.90y.o.(average) Clinical population no random sampling	EDE, EDI-P, CET	-compulsive exercise associated with perfectionism and ED In underweight adolescents; -compulsive exercise partially mediates the association between perfectionism and ED
Forbes et al., 2022 (38) USA	Longitudinal study to develop a self-report measure to assess the body-focused self-damaging behaviors frequency, co-occurrence, and related individuals’ average expectancies	Self-damaging behaviors, Disordered eating, Body-focused repetitive behaviors	Sample 1 Participants: 349 ♂88 ♀261 Age: 19.4 (average) Sample 2 Participants: 584 ♂204 ♀334 Age: 39 (average)	BSBEQ	Co-occurrence of body-focused self-damaging behaviors, shared expectancies across these behaviors.
Reinboth et al, 2022 (39) Norway	Cross-sectional study to investigate difference in motivational regulations for exercise and their predict potential on symptoms of EA and body image concerns	body image, exercise dependence, group fitness instructors, motivational regulations	Participants: 837 fitness trainer ♂152. Age: 38y.o (average) ♀685 Age: 33y.o. (average)	SIMS, EDS, EDI-DT, EDI-BD	External regulation predicts EA, drive for thinness, and body dissatisfaction.
Yildiz et al., 2022 (40) Turkiye	Longitudinal study to determine SMA effect on nutrition and exercise behavior in female adolescents	Adolescent, SMU, exercise, nutrition	Participants: 450 ♀450 Age 16.4y.o. (average)	SMAS-SF, NEBS	63.4% SMA positive correlation between SMAS-SF and psychological/addicted eating behavior and unhealthy diet-exercise behavior
Ahorsu et al., 2023 (41) China, USA, Taiwan, Sweden, Iran	Longitudinal study to investigate 1) the relationships between EA, insomnia, body image concerns, psychological distress and ED 2) the mediating roles of psychological distress, insomnia, and body concern between EA and ED	ED, EA, body image, coping	Participants: 2,088 ♂989 ♀1,099 Age 15.3y.o. (average)	DASS-21, EAI-Y, ISI, PSQI, BICI, EAT-26	Positive relationships between EA, psychological distress, insomnia, body image concern, and ED with small to large effect sizes
Mader et al., 2023 (42)​ Germany	Analysis of the relationship between SNS-usage, eating, and exercise behavior and their predictors	SNUD; ED; EA	Participants: 122 ♀51 ♂71 Age: 12–61y.o. (range)	SMD-scale, EDE-Q, EDS-21	2.5% EA (♀1.4%; ♂3.9%), 40.2% symptomatic non-dependent group (♀ 45.1%; ♂ 33.3%) 57.4% asymptomatic non-dependent (♀ 53.5%; ♂62.7%) 4.1% > the threshold for dysfunctional eating behavior (♀4.2%; ♂4.0%).
Morningstar et al., 2023 (43)​ Canada	Cross-sectional study to assess the association of the SMU profiles with the engagement in different domains of PA and the compliance to exercise recommendations	SMU profiles Five domains of physical activity (school curriculum, organized sport, exercise, outdoor play, and active transport)	Participants: 12,358 ♂5,648 ♂6,710 Age: adolescents	Data from the Canadian 2017/2018 Health Behavior in School-aged Children survey SMD-scale PA items	Non-active SMU was linked to lower probabilities of meeting the daily PA recommendations and of high engagement in all the five PA domains compared to active SMU. Intense SMU was associated with higher probability of meeting the daily PA recommendations. Problematic SMU was only significantly associated with lower odds of high PA engagement in the exercise domain
Tullet-Prado et al., 2023 (44)​ Australia	Survey research to assess type/structure of SMA symptoms and the association with gaming, gambling, alcohol, smoking, drug abuse, sex, shopping, internet use, and EA behaviours	SMA, Gaming, Gambling, Alcohol, Tobacco, IA, EA, SA, Shopping Addiction	Participants: 968 ♂622 ♀315 trans/non-binary: 26 queer 1 other 1 Age: 18–64y.o. (range)	BSMAS, IGDS-SF9, IDS9-SF, AUDIT, CDS-5; DAST-10, BYSAS, BSAS, EAI-R	High SMA risk: internet and shopping addictive behaviors, Moderately SMA risk: gaming, SA and gambling Weak SMA risk: alcoholism, substance abuse and EA
Tang et al., 2023 (45) USA	Survey research to assess the relationship between high EA risk and OCPD traits and higher levels of self-efficacy and correlation between higher OCPD traits and higher self-efficacy and EA	OCPD traits: obsessions, compulsions	Participants: 1,228 college students ♂606 ♀622 Age: 18–25y.o. (range)	EAI-R; GSES, PDQ-4+,	A higher prevalence of OCPD traits and self-efficacy were predictive of a greater risk of EA. Males were more prone at risk of EA. Higher prevalence of OCPD traits was significantly related to a greater risk of EA, but only at high levels of self-efficacy. Self-efficacy can moderate the relationship between OCPD traits and EA especially in females

♂, male gender; ♀, female gender; AUDIT, 10-Item Alcohol Use Disorders Identification Test; BD, body dissatisfaction, BDI, Beck Depression Inventory; BICI, Body Image concern Inventory; BYSAS, Bergen-Yale Sex Addiction Scale; BSAS, Bergen Shopping Addiction Scale; BSMAS, Bergen Social Media Addiction Scale; BSQ, Body Shape Questionnaire; CDS-5, the Five Item Cigarette Dependance Scale; CES, Compulsive Eating Scale; CET, Compulsive Exercise Test; CTQ, Childhood Trauma Questionnaire; DASS-21, Depression Anxiety Stress Scale 21 item; DAST-10, the 10- item Drug Abuse Screening Test; EA, Exercise Addiction; EAI, Exercise Addiction Inventory; EAI-R, 6-item Revised Exercise Addiction Inventory; EAI-Y, Exercise Addiction Inventory- Youth version; EAT-26, Eating Attitudes Test 26 item; ED, Eating Disorder; EDI-BD, Eating Disorders Inventory 3 - Body Dissatisfaction subscale; EDI-DT, Eating Disorders Inventory 3 - Drive for Thinness subscale; EDI-P Eating Disorders Inventory 3 - perfectionism subscale; EDE-Q, Eating Disorder Examination Questionnaire; EDS, Exercise Dependence Scale; EDS-21, The Exercise Dependence Scale Revised; EMS, Early Maladaptive Schema; FA, Food Addiction; FESA, Questionnaire to diagnose exercise dependence in endurance sports; GSES, General Self-Efficacy Scale; HASE, Homburger ADHS Skalen für Erwachsene; IA, Internet Addiction; IDS9-SF, The Internet Disorder Scale; IGDS-SF9, Internet Gaming Disorder 9 items Short Form; ISI, Insomnia Severity Index; MIPS, The multidimensional inventory of perfectionism in sport; NEBS, Nutrition Exercise Behavior Scale; NEO-PI-R, NEO Personality Inventory Revised; OCPD, obsessive compulsive personality disorder; PA, Physical Activity; PDQ-4+, 8-item OCPD subscale in the self-report Personality Diagnostic Questionnaire-4thEdition Plus; PSMU, Problematic social media use; SA, Sex Addiction; SAPAS, Standardized Assessment of Personality Abbreviated Scale; SCID-5, Structured Clinical Interview for DSM-5; SDE, Scale of dedication to exercise; SE-IAT, The Implicit Association Test for self-esteem; SMA, Social Media Addiction; SMAS-SF, Social Media Addiction Scale for Adolescents; SMU, Social Media Use; y.o, years old; YFAS 2.0, Yale food addiction scale 2.0; YSQ-S3, Young Schema Questionnaire Short Form Revised.

### 3.2 Results in domain investigated

The 15 studies included (**Table 1**) in the literature search considered 4 main domains, such as body image-related dysfunction, eating disorders, difficult individual traits, and/or problematic social media use. As a result, different connections with EA emerged.

Two different studies highlighted the strong association between EA and ED which have in common weight concerns, perfectionism, perception of body image, body dissatisfaction, depression, psychological distress and insomnia (32, 41). Furthermore, compulsive exercise plays a role in mediating the clinical perfectionism and EA, especially in vulnerable athletes or underweight adolescents (31, 37).

Body dissatisfaction (BD) is a disorder characterized by individual suffering due to the difference between what is the real and the idealistic image of the body. It has been reported that BD is a risk factor for the development of EA and ED especially in fitness instructors or practitioners (32, 39). More recently, cognitive constructs were investigated in relation to EA. Indeed, the relationship between Early Maladaptive Schema (EMS) and EA, the only two specific domains which influenced EA were the other-directedness and the impaired limits. To this concern, individuals unable to set appropriate internal limits and have excessive external focus on others’ desires and needs may be more prone to developing EA (33).

Recently, the EA was investigated in relation to PSMU consequences on mental health, focusing on anxiety, depression, and stress rather than personality traits such as extraversion, perfectionism, and aggression. As a result, EA appeared to play a mediating role since it is strictly connected to body image concerns, psychological distress and compulsive eating, which may cause negative mental health outcomes influencing the PSMU (35).

SMA was positively correlated with psychological/addicted eating behavior and unhealthy diet-exercise behavior, but negatively with healthy eating/exercise behavior (40). SMU impacts the physical activity behavior. The passive SMU corresponds to a low rate of daily physical activity practice (minimum 60 minutes), while the actively SMU is linked to a higher probability of exercise activity (43). On the other hand, another study reported that EA is not associated with the frequency of active or passive social networking sites usage (42)

SMA may be part of a broader spectrum of addictive behaviors. It was found that EA and substance abuse are weakly related to SMA while this latter is significantly associated with shopping addiction (44).

The relationship between EA, depression, and anxiety has been extensively studied, indeed many studies reported that EA co-occurs with mental health disorders such as major depressive disorder, anxiety, obsessive-compulsive personality disorder (34, 36, 45). Moreover, people with obsessive-compulsive traits and high levels of self-efficacy are at higher risk of becoming exercise addicts (45).

## 4 Discussion

Whereas different EA definitions have been formulated, the terminology is still inconsistently used with “exercise addiction” and “compulsive exercise” or “exercise dependence” used as synonyms, although labile characteristics allow to discriminate the different conditions related to excessive exercise. A panel of experts in the field, including physicians, physiotherapists, coaches, trainers, and athletes defined excessive exercise as an “addiction”, identifying perfectionism, obsessive-compulsive drive, and hedonism as components of EA (23). The co-occurrence of these components and the excessive exercise was described as a behavioral addiction with a similar mechanism to substance addictions (23).

In recent years, the scientific community raised concerns about the connection between EA, SMU, and mental health, due to the potential outcomes on mental well-being of the recent spreading of both excessive exercise and extensive social media. Among the others, the complex relationship between EA, and SMA, perfectionism, body image disorders, and “fitspiration” construct have been studied, since 2015 (46). Interestingly, “fitspiration” diversion into a distortion of body perception could emerge (20, 47, 48). Although “fitspiration” generally conveys positive messages, the images associated with that may have negative effects on the body image of individuals who engage in it, as they predominantly portray a lean and toned body figure. Noteworthy, “fitspiration” emerged as a positive alternative to the “thin-spiration” trend, combining “thin” and “inspiration”. Notably, perfectionism is a personality trait in individuals characterized by unrealistic expectations for themselves and others, with feelings of inadequacy, self-criticism, and guilt (49), with different mechanisms of connection to EA and ED, although there is still limited clarity on EA mediating role between perfectionism and ED. Furthermore, EA is related to other personality traits such as the tendency towards depression, or an inability to manage it. These traits also constitute risk factors for behavioral addictions, such as SMA, which also involves a distortion of body perception. The EA appears to have a similar developmental pattern to other addiction or addictive behaviors, following the biopsychosocial model (50). Besides, EA has consistent co-occurrence patterns to depression and anxiety revealing that individuals with obsessive-compulsive traits and high self-efficacy present high risk of becoming exercise addicted.

However, a lack of specific and robust tools to study the EA emerged, imposing the adaptation of diagnostic tools validated for the assessment of other behavioral addictions such as gambling or gaming disorders. Hence, certain diagnostic criteria are still not provided to clinicians to precisely identify the EA. Otherwise, people are widely informed through social media communications about the EA associated risks. Furthermore, the lack of specific tools determines that only the relation with other disorders is evaluated, while EA is never considered alone. Indeed, the EA role as a consequence or cause of a broad spectrum of other disorders should be clarified.

On the other hand, study protocols should be harmonized, preferably based on standardized measurement tools that would ensure result consistency. This approach would facilitate the study of EA, clarifying the mediating role of behavioral addictions on mental health.

A general weakness in EA investigation is represented by the size and the quality of the population involved in the studies. Indeed, only four studies considered at least 1,000 individuals (31, 35, 41, 45), and only one investigated over 10,000 participants (43). Moreover, the adult population were the most examined while only one-third of the studies included the adolescent population. To this concern, the adolescent population should be further explored, considering the early onset of behavioral addictions, and ED (51–54).

Lastly, another important issue is the lack of validated intervention and prevention programs with evidence-based efficacy in managing EA.

## 5 Conclusion

The relationship between EA, SMU, and mental health is intricate and knotted. While both exercise and social media have the potential to contribute positively to mental well-being, their excessive and addictive use can lead to adverse outcomes. Improved knowledge on mechanisms and assessment of individual differences are essential to develop effective interventions, promoting healthy exercise habits and mindful social media use. Eventually, improved mental health and well-being in the digital age would be fostered. Finally, it would be necessary to expand the number of these studies to identify risk factors and protective factors, which in turn are fundamental elements for implementing prevention strategies for behavioral addictions.

## Author contributions

AM: Conceptualization, Data curation, Formal analysis, Investigation, Methodology, Project administration, Resources, Supervision, Writing – original draft, Writing – review & editing. AT: Data curation, Formal analysis, Investigation, Methodology, Writing – original draft, Writing – review & editing. VA: Validation, Writing – review & editing. GC: Data curation, Formal analysis, Investigation, Methodology, Writing – original draft. PB: Writing – review & editing. NM: Data curation, Investigation, Methodology, Visualization, Writing – original draft, Writing – review & editing.
